# Adjunctive long-acting risperidone in patients with bipolar disorder who relapse frequently and have active mood symptoms

**DOI:** 10.1186/1471-244X-11-171

**Published:** 2011-10-28

**Authors:** Wayne Macfadden, Caleb M Adler, Ibrahim Turkoz, John T Haskins, Norris Turner, Larry Alphs

**Affiliations:** 1 Formerly, Janssen Scientific Affairs, LLC, Titusville, NJ, USA; 2 University of Cincinnati College of Medicine, Cincinnati, OH, USA; 3 Johnson & Johnson Pharmaceutical Research and Development, LLC, Titusville, NJ, USA; 4 Janssen Scientific Affairs, LLC, Titusville, NJ, USA

## Abstract

**Background:**

The objective of this exploratory analysis was to characterize efficacy and onset of action of a 3-month treatment period with risperidone long-acting injection (RLAI), adjunctive to an individual's treatment regimen, in subjects with symptomatic bipolar disorder who relapsed frequently and had significant symptoms of mania and/or depression.

**Methods:**

Subjects with bipolar disorder with ≥4 mood episodes in the past 12 months entered the open-label stabilization phase preceding a placebo-controlled, double-blind study. Subjects with significant depressive or manic/mixed symptoms at baseline were analyzed. Significant depressive symptoms were defined as Montgomery-Åsberg Depression Rating Scale (MADRS) ≥16 and Young Mania Rating Scale (YMRS) < 16; manic/mixed symptoms were YMRS ≥16 with any MADRS score. Subjects received open-label RLAI (25-50 mg every 2 weeks) for 16 weeks, adjunctive to a subject's individualized treatment for bipolar disorder (mood stabilizers, antidepressants, and/or anxiolytics). Clinical status was evaluated with the Clinical Global Impressions of Bipolar Disorder-Severity (CGI-BP-S) scale and changes on the MADRS and YMRS scales. Within-group changes were evaluated using paired *t *tests; categorical differences were assessed using Fisher exact test. No adjustment was made for multiplicity.

**Results:**

162 subjects who relapsed frequently met criteria for significant mood symptoms at open-label baseline; 59/162 (36.4%) had depressive symptoms, 103/162 (63.6%) had manic/mixed symptoms. Most subjects (89.5%) were receiving ≥1 medication for bipolar disorder before enrollment. Significant improvements were observed for the total population on the CGI-BP-S, MADRS, and YMRS scales (p < .001 vs. baseline, all variables). Eighty-two (53.3%) subjects achieved remission at the week 16 LOCF end point. The subpopulation with depressive symptoms at open-label baseline experienced significant improvement on the CGI-BP-S and MADRS scales (p < .001 vs. baseline, all variables). Subjects with manic/mixed symptoms at baseline had significant improvements on the CGI-BP-S and YMRS scales (p < .001 vs. baseline, all variables). No unexpected tolerability findings were observed.

**Conclusions:**

Exploratory analysis of changes in overall clinical status and depression/mania symptoms in subjects with symptomatic bipolar disorder who relapse frequently showed improvements in each of these areas after treatment with RLAI, adjunctive to a subject's individualized treatment. Prospective controlled studies are needed to confirm these findings.

## Background

Bipolar disorder is a serious, lifelong mental illness associated with marked psychosocial disability [1-5]. Although the goal of treatment during an acute episode is symptom control to preserve psychosocial functioning [6], patients with bipolar disorder who relapse frequently are a difficult-to-treat population [7,8]. In many cases, clinicians may initiate treatment with monotherapy; however, therapeutic management often requires the addition of adjunctive medications that can include mood stabilizers, antidepressants, or antipsychotics [6].

A significant barrier to treatment of bipolar disorder is nonadherence. In a sample of outpatients, 24% of subjects were found to be at least partially nonadherent on 20% or more of study visits [9]. Factors that have been associated with poor adherence include history of rapid cycling, bipolar type I disorder, and greater illness severity [9,10]. Poor adherence to medication has been associated with a higher rate of recurrence and hospitalization [11,12]. Subjects who were adherent at least 75% of the time were at lower risk for all-cause rehospitalization and mental health-related rehospitalization [12]. Therefore, improving adherence is likely to result in improved treatment outcomes.

Oral antipsychotics are often used adjunctively to treat the symptoms of bipolar disorder, but their effectiveness may be compromised by poor medication adherence. Long-acting injectable atypical antipsychotics may allow clinicians to identify and respond more easily to poor adherence [13]. One long-term, prospective study of acutely manic inpatients with bipolar disorder and a history of poor or partial adherence found that risperidone long-acting injection (RLAI) significantly decreased hospitalization rates and reduced discontinuation of all medications the patients were taking [14]. Further, RLAI as maintenance therapy has been observed to significantly delay time to relapse in subjects with bipolar disorder when used either as monotherapy or as an adjunct to individualized pharmacotherapy in subjects who relapse frequently [15,16].

The objective of this post hoc analysis was to examine clinical, symptomatic, and functional outcomes during the 16-week, open-label phase of an international (United States and India), double-blind, relapse-prevention study examining the addition of adjunctive RLAI to individualized pharmacotherapy in subjects with bipolar disorder who relapsed frequently over the previous 12 months (NCT00094926) [15]. The aim of the analysis was to determine whether the addition of RLAI to individual treatment regimens of mood stabilizers, antidepressants, and/or anxiolytics was beneficial in a subset of subjects from this study who were experiencing depressive or manic/mixed symptoms.

## Methods

### Study Design

This post hoc analysis examined data from the 16-week, open-label stabilization phase that preceded the randomized, double-blind, relapse-prevention phase. The protocol was approved by an institutional review board or ethics committee at each site, and the study was conducted in accordance with current International Conference on Harmonization/World Health Organization Good Clinical Practice guidelines and the Declaration of Helsinki.

### Subjects

Eligible subjects were 18-70 years of age, had bipolar type I or II disorder, diagnosed using the *Diagnostic and Statistical Manual of Mental Disorders*, Fourth Edition, Text Revision, and had experienced 4 or more mood episodes requiring psychiatric intervention in the previous 12 months [15]. In the original study, subjects with any degree of mood symptom severity were included. The current analysis focused only on subjects with significant depressive or manic/mixed symptoms at open-label baseline (depressive symptoms: Montgomery-Åsberg Depression Rating Scale [MADRS] [17] ≥16 and Young Mania Rating Scale [YMRS] [18] < 16; manic/mixed symptoms: YMRS ≥16 with any MADRS score).

### Treatment

RLAI 25 mg every 2 weeks was initiated at open-label baseline, with optional dosage increases to 37.5 mg at week 4 and to 50 mg at week 10 (per the investigators' clinical judgment). Oral antipsychotics that subjects were taking before the study were continued for 3 weeks after the first RLAI injection, and subjects who were not taking oral antipsychotics received oral risperidone. Additional medications for bipolar disorder were individually determined for each subject and could include any number or combination of antidepressants, mood stabilizers, and anxiolytics, with the exception of carbamazepine, oxcarbazepine, fluoxetine, and paroxetine. These medications were initiated, resumed, or changed at the discretion of the investigators at any time during the first 12 weeks of open-label stabilization.

### Assessments

Clinical status was determined by the Clinical Global Impressions of Bipolar Disorder-Severity (CGI-BP-S) scale [19];manic and depressive symptoms were measured using the YMRS and MADRS, respectively. Assessments were performed at baseline and weeks 4, 8, 12, and 16. Remission was defined as YMRS total score ≤8, MADRS total score ≤10, and CGI-BP-S score ≤2. Functioning was assessed by the Global Assessment of Functioning (GAF) scale [20], conducted at baseline and at week 16. Scores on the GAF scale range from 0 to 100, with higher scores indicating better functioning. Safety was determined by adverse event (AE) monitoring at each visit.

### Statistical Analysis

Efficacy and safety outcomes were analyzed in subjects enrolled in the open-label phase who received ≥1 dose of RLAI. Demographic and baseline characteristics were summarized using descriptive statistics. Last-observation-carried-forward (LOCF) methodology was used for the YMRS, MADRS, CGI-BP-S, and GAF analyses at end point. Subjects completing 16 weeks of treatment (completers) also were evaluated. A change of ≥10 points in the GAF score also was identified. Within-group changes from open-label baseline were evaluated using paired *t *tests; categorical differences were assessed by Fisher exact test. All statistical tests were 2-sided, and the nominal type I error was fixed at 0.05. No adjustments were made for multiplicity.

## Results

### Baseline Demographics, Clinical Characteristics, and Disposition

One hundred sixty-two (58.9%) of the 275 subjects who enrolled in the original study had significant mood symptoms at open-label baseline. Of the 162 subjects, 59 (36.4%) subjects had significant depressive symptoms and 103 (63.6%) had significant manic/mixed symptoms (Table 1). Of the subjects with current depressive symptoms, 81.4% were diagnosed with bipolar type I disorder, as were 93.2% of subjects with manic/mixed symptoms. A higher percentage of symptomatic women (44.3%) than symptomatic men (30.4%) had significant depressive symptoms; 69.6% of symptomatic men and 55.7% of symptomatic women had significant manic/mixed symptoms. The most recent episode for 74.6% of subjects with significant current depressive symptoms was a depressive episode; the most recent episode for 70.9% of subjects with significant manic/mixed symptoms was a manic episode. Overall, 74.1% of subjects with significant mood symptoms completed the 16-week open-label phase: 74.6% subjects with depressive symptoms and 73.8% with manic/mixed symptoms (Table 1).

**Table 1:** Baseline demographic and clinical characteristics, disposition, and RLAI mean daily dose and dose distribution (ITT analysis set)

	Total (N = 162)	Baseline Depressive Symptoms (n = 59)	Baseline Manic or Mixed Symptoms (n = 103)
**Baseline demographic and clinical characteristics**			
Age, years			
Mean (SD)	38.6 (11.4)	41.0 (11.5)	37.2 (11.2)
Median (range)	39 (18-70)	41 (22-70)	38 (18-61)
Gender, n (%)			
Male	92 (56.8)	28 (47.5)	64 (62.1)
Female	70 (43.2)	31 (52.5)	39 (37.9)
Race, n (%)			
Caucasian	51 (31.5)	19 (32.2)	32 (31.1)
Hispanic	3 (1.9)	2 (3.4)	1 (1.0)
Black	18 (11.1)	5 (8.5)	13 (12.6)
Other (Indian)	90 (55.6)	33 (55.9)	57 (55.3)
Bipolar disorder subtype, n(%)			
Type I	144 (88.9)	48 (81.4)	96 (93.2)
Type II	18 (11.1)	11 (18.6)	7 (6.8)
Most recent episode, n (%)			
Depressed	56 (34.6)	44 (74.6)	12 (11.7)
Manic	79 (48.8)	6 (10.2)	73 (70.9)
Mixed	19 (11.7)	8 (13.6)	11 (10.7)
Hypomanic	8 (4.9)	1 (1.7)	7 (6.8)
Time since most recent episode, weeks, mean (SD)	6.0 (4.6)	6.1 (4.8)	5.9 (4.5)
**Disposition**			
Completed OL phase	120 (74.1)	44 (74.6)	76 (73.8)
Discontinued	42 (25.9)	15 (25.4)	27 (26.2)
Reason for discontinuation			
Withdrawal of consent	14 (8.6)	7 (11.9)	7 (6.8)
AEs	13 (8.0)	6 (10.2)	7 (6.8)
Lost to follow-up	10 (6.2)	2 (3.4)	8 (7.8)
Nonadherent	1 (0.6)	0 (0)	1 (1.0)
Other	4 (2.5)^a^	0 (0)	4 (3.9)^a^
**RLAI mean daily dose and dose distribution**			
Dose, mg, mean (SD)	27.9 (5.6)	26.5 (4.1)	28.6 (6.2)
Dose distribution, n (%)			
25 mg	127 (78.4)	52 (88.1)	75 (72.8)
37.5 mg	33 (20.4)	7 (11.9)	26 (25.2)
50 mg	2 (1.2)	0 (0)	2 (1.9)

OL, open-label; RLAI, risperidone long-acting therapy; SD, standard deviation.

^a^Of these subjects, 3 discontinued because of lack of efficacy and 1 because of pregnancy.

### Bipolar Disorder Medication Use and RLAI Dose

Most subjects in the total symptomatic population at baseline (89.5%) were taking ≥1 medication for bipolar disorder before enrollment; 47 (29.0%) were receiving oral antipsychotics. For subjects who completed 16 weeks of treatment, with the exception of a higher use of antidepressants compared with baseline (42.5 vs. 29.6%), the number of medications taken for bipolar disorder was generally similar at baseline and week 16 (Table 2).

**Table 2:** Bipolar disorder medications (ITT population)

OL Baseline			
	Total (N = 162)	Baseline Depressive Symptoms (n = 59)	Baseline Manic/Mixed Symptoms (n = 103)
**Number of bipolar medications^a, b^**			
0	17 (10.5)	5 (8.5)	12 (11.7)
1	37 (22.8)	14 (23.7)	23 (22.3)
2	49 (30.3)	16 (27.1)	33 (32.0)
≥3	59 (36.4)	24 (40.7)	35 (34.0)
Mood stabilizers	128 (79.0)	44 (74.6)	84 (81.6)
Antipsychotics	47 (29.0)	17 (28.8)	30 (29.1)
Antidepressants	48 (29.6)	26 (44.1)	22 (21.4)
Anxiolytics	54 (33.3)	20 (33.9)	34 (33.0)
**OL Week 16 (Completers)**			
	**Total** **(N = 120)**	**Baseline** **Depressive** **Symptoms** **(n = 44)**	**Baseline** **Manic or Mixed Symptoms** **(n = 76)**
**Number of bipolar disorder medications^a, c^**			
0	8 (6.7)	0 (0)	8 (10.5)
1	41 (34.2)	16 (36.4)	25 (32.9)
2	39 (32.5)	17 (38.6)	22 (29.0)
≥3	32 (26.7)	11 (25.0)	21 (27.6)
Mood stabilizers	99 (82.5)	35 (79.6)	64 (84.2)
Antidepressants	51 (42.5)	28 (63.6)	23 (30.3)
Anxiolytics	32 (26.7)	11 (25.0)	21 (27.6)

ITT, intent to treat; OL, open-label.

^a^A subject taking > 1 medication within a class and subclass was counted once within the class and subclass.

^b^Classes included mood stabilizers, antidepressants, antipsychotics, and anxiolytics.

^c^Classes included mood stabilizers, antidepressants, and anxiolytics.

Of depressive and manic/mixed subjects, 91.5% and 88.3% at baseline, respectively, were taking ≥1 medication; similar proportions of subjects were taking antipsychotics. At week 16, with the exception of a higher use of antidepressants compared with baseline for the depressive population (63.6% vs. 44.1%), the number of medications received for bipolar disorder was generally similar at baseline and week 16.

The median dose of RLAI during the open-label stabilization phase for all symptomatic subjects was 25 mg every 2 weeks; the mean doses and the dose distributions for the depressive and manic/mixed groups were similar (Table 1).

### Total Population of Subjects with Significant Mood Symptoms

#### Efficacy

**Figure 1:**
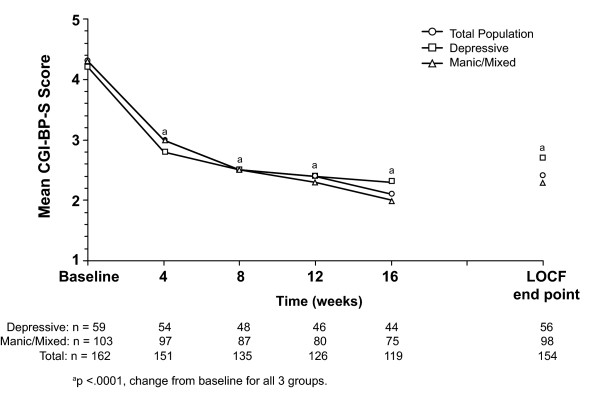
**Mean CGI-BP-S score over time (ITT analysis set)**. CGI-BP-S, Clinical Global Impressions of Bipolar Disorder-Severity; LOCF, last observation carried forward.

**Figure 2:**
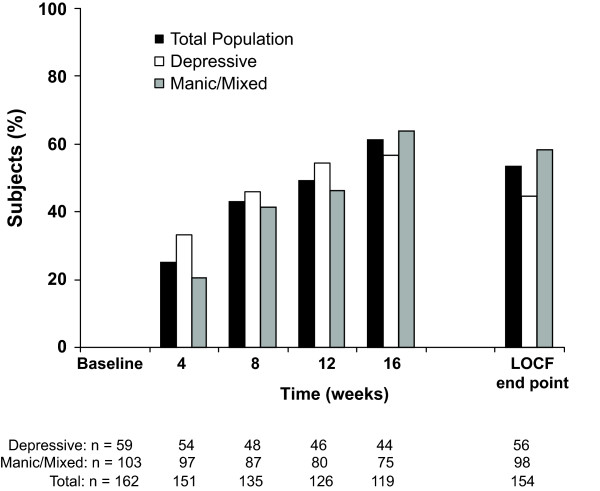
**Point remission rates**. LOCF, last observation carried forward (ITT analysis set).

The clinical status improved significantly by week 4 and at each subsequent time point, as determined by CGI-BP-S total scores (Figure 1). Mood symptoms also improved, as reflected in significant decreases in mean MADRS and YMRS scores for subjects at LOCF end point and for completers (Table 3). Remission was attained by 53.3% of subjects at LOCF end point and by 61.3% of completers (Figure 2). A 10-point improvement in GAF score was observed in 62.3% of subjects at LOCF end point and in 68.9% of completers, with mean (standard deviation [SD]) GAF scores improving 16.3 (17.1; p < .001) points and 19.0 (16.7; p < .001) points, respectively (Table 3).

**Table 3:** Efficacy measures: baseline and end point values in the open-label stabilization phase (ITT population)

	Total (N = 162)	Baseline Depressive Symptoms (n = 59)	Baseline Manic or Mixed Symptoms (n = 103)
**CGI-BP-S, mean (SD)**			
Baseline	4.3 (0.8)	4.2 (0.8)	4.3 (0.8)
Change from baseline			
Completers^a^	-2.1 (1.4)^e^	-1.9 (1.5)^e^	-2.2 (1.3)^e^
LOCF end point^b^	-1.8 (1.5)^e^	-1.5 (1.6)^e^	-2.0 (1.5)^e^
**MADRS, mean (SD)**			
Baseline	15.0 (11.6)	25.4 (6.4)	9.0 (9.5)
Change from baseline			
Completers^a^	-7.2 (11.2)^e^	-15.6 (10.7)^e^	-2.2 (8.1)^d^
LOCF end point^b^	-6.0 (12.6)^e^	-14.0 (11.2)^e^	-1.4 (11.0)
**YMRS, mean (SD)**			
Baseline	18.8 (12.1)	5.8 (4.5)	26.3 (8.2)
Change from baseline			
Completers^a^	-14.2 (12.5)^e^	-2.3 (5.5)^d^	-21.2 (9.9)^e^
LOCF end point^b^	-13.2 (13.8)^e^	-0.9 (8.6)	-20.2 (11.1)^e^
**GAF**			
≥10-point improvement (%)^e^			
Completers^a^	68.9	63.6	72.0
LOCF end point^c^	62.3	56.4	65.9
Baseline, mean (SD)	50.9 (11.4)	52.5 (10.0)	49.9 (12.1)
Change from baseline, mean (SD)			
Completers^a^	19.0 (16.7)^e^	15.5 (16.4)^e^	21.0 (16.7)^e^
LOCF end point^c^	16.3 (17.1)^e^	13.0 (16.6)^e^	18.4 (17.1)^e^

CGI-BP-S, Clinical Global Impressions of Bipolar Disorder-Severity; GAF, Global Assessment of Functioning; ITT, intent to treat; LOCF, last observation carried forward; MADRS, Montgomery-Åsberg Depression Rating Scale; SD, standard deviation; YMRS, Young Mania Rating Scale.

^a^n = 119, n = 44, and n = 75; ^b^n = 154, n = 56, and n = 98; ^c^n = 146, n = 55, and n = 91 for the total, depressive, and manic/mixed populations, respectively.

^d^p < .05 vs. baseline; ^e^p < .001 vs. baseline.

#### Safety

Safety results were similar to those previously reported [15]. Most (75.3%) subjects experienced ≥1 AE during this 16-week period. The most common AEs, with an incidence of ≥10%, were tremor (22.8%), muscle rigidity (15.4%), weight increase (13.6%), and headache (11.1%). Eight percent of the population discontinued because of AEs. At baseline, the mean (SD) weight was 74.1 (20.4) kg. The mean (SD) weight increase from baseline was 2.0 (4.1) kg for subjects at LOCF end point and 2.1 (4.4) kg for completers (p < .001 vs. baseline for both comparisons).

### Subjects with Depressive Symptoms at Baseline

#### Efficacy

**Figure 3:**
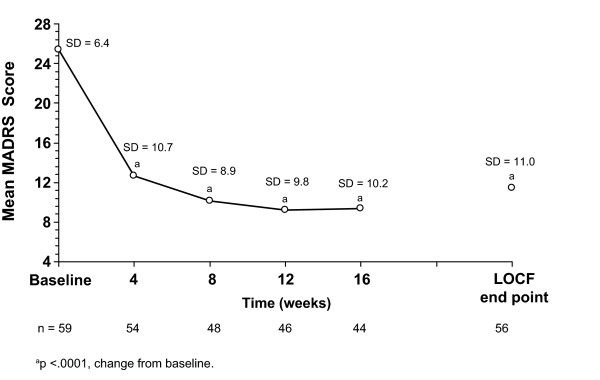
**Mean MADRS scores for subjects with depressive symptoms at open-label baseline (ITT analysis set)**. MADRS, Montgomery-Åsberg Depression Rating Scale; LOCF, last observation carried forward; SD, standard deviation.

Mean scores significantly improved on the CGI-BP-S by week 4 and each subsequent time point (Figure 1). Remission was achieved by 44.6% of subjects at LOCF end point and by 56.8% of completers (Figure 2). Mean MADRS scores decreased significantly at each time point including LOCF end point (Figure 3). A 10-point improvement in GAF score was observed in 56.4% of subjects at LOCF end point and in 63.6% of completers, and the mean (SD) change from baseline in GAF scores was 13.0 (16.6) (p < .001) and 15.5 (16.4) (p < .001), respectively (Table 3).

#### Safety

The proportion of subjects who had ≥1 AE was 69.5%. The most common AEs, with an incidence of ≥10%, were tremor (17.0%), headache (13.6%), muscle rigidity (11.9%), fatigue (11.9%) and somnolence (10.2%). The proportion of subjects who discontinued because of AEs was 10.2%. At baseline, the mean (SD) weight was 72.5 (21.2) kg. The mean (SD) weight increase from baseline was 2.5 (3.8) kg for subjects at LOCF end point and 2.7 (3.9) kg for completers (p < .001 vs. baseline for both comparisons).

### Subjects with Manic/Mixed Symptoms at Baseline

#### Efficacy

**Figure 4:**
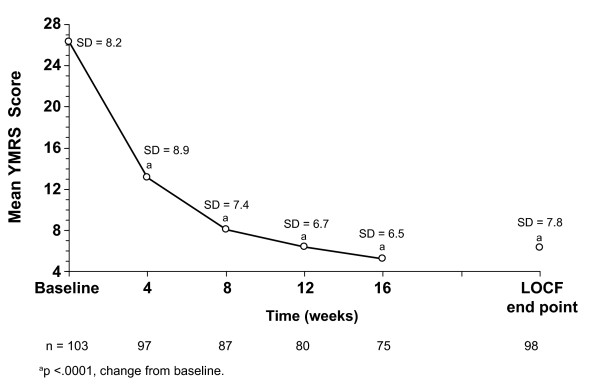
**Mean YMRS scores for subjects with manic/mixed symptoms at open-label baseline (ITT analysis set)**. LOCF, last observation carried forward; SD, standard deviation; YMRS, Young Mania Rating Scale.

The overall clinical status of subjects with manic/mixed symptoms at baseline also significantly improved, as seen in significant changes on the CGI-BP-S at each time point, again starting at Week 4, including LOCF end point (Figure 1). Remission was attained by 58.2% of subjects at LOCF end point and by 64.0% of completers (Figure 2). Mean YMRS scores decreased significantly at each time point, including LOCF end point (Figure 4). There was a small but significant improvement in MADRS scores for completers (p < .05). No significant improvement was observed in subjects at LOCF end point (Table 3). A 10-point improvement in GAF score was observed in 65.9% of subjects at LOCF end point and in 72.0% of subjects who completed the study, and the mean (SD) improvement from baseline in GAF scores was 18.4 (17.1) and 21.0 (16.7) (p < .001), respectively (Table 3).

#### Safety

At least 1 AE was observed in 78.6% of subjects experiencing manic/mixed symptoms. The most common AEs with an incidence of ≥10% were tremor (26.2%), muscle rigidity (17.5%), weight increase (17.5%), and sedation (11.7%); 6.8% of subjects discontinued because of AEs. At baseline, the mean (SD) weight was 75.1 (20.0) kg. The mean (SD) weight increase from baseline was 1.8 (4.3) kg for subjects at LOCF end point and 1.8 (4.6) kg for completers (p < .01 vs. baseline for both comparisons).

## Discussion

The efficacy and safety of maintenance RLAI as monotherapy or adjunctive therapy in subjects with bipolar disorder have been confirmed in large, controlled studies [15,16,21]. However, the particular types of patients with bipolar disorder who might best be considered for this treatment have not been fully established. Data from this post hoc analysis suggest that patients with a history of frequent relapse who experience acute symptoms might benefit from the addition of RLAI to their current treatment regimen of mood stabilizers, antidepressants, and/or anxiolytics.

Because the data reported here represent a post hoc evaluation, these results are specific to the population studied here and may not be readily generalizable to the broader population of patients with bipolar disorder. A substantial proportion of subjects entered the relapse-prevention study with significant symptoms, despite receiving bipolar disorder medications at baseline. This may support the fact that this frequently-relapsing population is difficult to manage and has poor adherence to medication. The addition of adjunctive RLAI was associated with significant improvements in clinical status and symptoms by week 4, as determined by the CGI-BP-S, YMRS, and MADRS scales. Remission was achieved in more than one-half of the total population by the 16-week LOCF end point and more than 60% had a 10-point improvement on the GAF scale.

Determining a medication's effectiveness in treating the manic and depressive symptoms of bipolar disorder is important for patient management. For subjects with manic/mixed symptoms, RLAI treatment resulted in clinical and symptom improvement within 4 weeks of treatment initiation with significant increases in remission rates and patient functioning. RLAI also was found to be effective in subjects with depressive symptoms. These patients are typically difficult to treat and are associated with poor functioning [22] and a high frequency of depressive episodes has been reported to be predictive of nonadherence [11]. In the current study, subjects with depressive symptoms showed significant clinical and symptom improvement by week 4 and a majority of subjects achieved remission. Also, more than half of the subjects with depressive and manic/mixed symptoms achieved a significant improvement in functioning. Although the analyses were not preplanned to analyze differences in efficacy/tolerability between these groups of subjects, these data may suggest that RLAI may be effective regardless of depressive or manic/mixed symptoms.

No unexpected safety or tolerability findings were identified, and AE rates were similar to those found in the overall study population [15]. This suggested that the tolerability profile may be independent of mood state or severity of symptoms. The mean weight of subjects increased by approximately 2 kg, whether measured at study end point or at completion of all 16 weeks of treatment.

Although they suggest that RLAI is effective in patients with bipolar disorder who have frequent relapses, these results must be interpreted with caution. As a post hoc analysis of an open-label stabilization phase of a relapse-presentation, this study did not include a control group. Nonetheless, the efficacy results observed in this post hoc analysis with RLAI were generally similar to those of the open-label stabilization phase of the overall study [15]. Also, subject compliance with their individual treatment regimens of mood stabilizers, antidepressants, and/or anxiolytics before study entry was not established. Therefore, improvements seen in this analysis may be due in part to noncompliance with previous medications. Additionally, due to the release profile of RLAI (< 1% of risperidone is released during the first 3 weeks) [23] oral supplementation with antipsychotics was required for the first 3 weeks of the study. This may have influenced the results at the earlier time points. However, by the week 4 assessment the main release of RLAI would have occurred per the prescribing information [23]. Remission was analyzed at each individual time point and did not account for a subject's remission status at previous time points during the open-label stabilization phase. Therefore, the percentage of subjects who met stable remission criteria could not be established. Nonetheless, over half of subjects met remission criteria by week 16. Although there may appear to be differences in onset of remission for the 2 subpopulations there were substantial between-group differences in baseline demographics, baseline disease characteristics, symptomatology, as well as the scales used to measure symptoms. While these data may be hypothesis-generating, the timing of improvement of symptom domains among these different subpopulations could not be established.

## Conclusions

To summarize, in subjects with symptomatic bipolar disorder who experienced frequent relapses, significant improvements were observed with regard to mood symptoms, clinical status, and functioning, after the addition of RLAI to their current treatment regimen of mood stabilizers, antidepressants, and/or anxiolytics. Remission was achieved by approximately one-half of all subjects during 16 weeks of treatment, with improvement observed as early as 4 weeks. Benefits were observed in subjects with depressive symptoms or manic/mixed symptoms. The addition of RLAI, therefore, may be useful for adjunctive treatment in patients with bipolar disorder who continue to frequently experience symptoms despite previous and ongoing treatment.

## Competing interests

At the time of this analysis, W Macfadden was a full-time employee of Janssen Scientific Affairs, LLC. L Alphs and N Turner are full-time employees of Janssen Scientific Affairs, LLC, and Johnson & Johnson stockholders. JT Haskins and I Turkoz are full-time employees of Johnson & Johnson Pharmaceutical Research and Development, LLC, and Johnson & Johnson stockholders. CM Adler over the last 12 months has received honoraria for speaking and consulting from Merck, as well as research support from Abbott Laboratories, AstraZeneca, Eli Lilly, Shire, Johnson & Johnson, Pfizer, Repligen, and Martek. With the exception of AstraZeneca, the research support has been in the form of payments for multisite clinical trials.

## Authors' contributions

WM, LA, IT, JTH, and NT contributed to the conception and design, acquisition of data, analysis and interpretation of data, and drafting of the manuscript and its critical revision for important intellectual content. CMA was involved in the interpretation of data and in the critical drafting and revising of the manuscript for important intellectual content. All authors read and approved the final manuscript.

## Pre-publication history

The pre-publication history for this paper can be accessed here:

http://www.biomedcentral.com/1471-244X/11/171/prepub
